# RETRACTED: African swine fever virus B175L inhibits the type I interferon pathway by targeting STING and 2′3′-cGAMP

**DOI:** 10.1128/jvi.00795-23

**Published:** 2023-10-30

**Authors:** Lakmal Ranathunga, Niranjan Dodantenna, Ji-Won Cha, Kiramage Chathuranga, W. A. Gayan Chathuranga, Asela Weerawardhana, Ashan Subasinghe, D. K. Haluwana, Nuwan Gamage, Jong-Soo Lee

**Affiliations:** 1 College of Veterinary Medicine, Chungnam National University, Daejeon, South Korea; Cornell University Baker Institute for Animal Health, Ithaca, New York, USA

**Keywords:** African swine fever virus (ASFV), B175L, 2′3′-cGAMP, STING and type I interferon

## Abstract

Type I interferon (IFN)-mediated responses operate a pivotal role in the first line of host immune defense against viruses. Upon DNA virus infection, DNA sensing cyclic GMP-AMP synthase-STING signal pathway is a key event in the IFN-mediated antiviral response and also a target of viral immune evasion. African swine fever (ASF) is a highly contagious, cross-border viral disease that often confers fatalities in domestic and feral pigs of all ages. African swine fever virus (ASFV), a causative agent of ASF, is a nucleocytoplasmic large DNA virus that masks critical elements of host immune system by several encoded proteins. In this study, we report a novel immune evasion mechanism of ASFV B175L to block type I IFN signaling by targeting STING and 2'3'-cyclic GMP-AMP (2′3′-cGAMP). We found that ASFV B175L significantly inhibited the DNA virus-induced IFN-β production and IFN-mediated signaling responses. The conserved zf-FCS motif of ASFV B175L competitively interacted with both cGAMP and the cyclic dinucleotide binding domain of STING. Remarkably, the R238 and Y240 amino acids of STING were crucial for interaction with ASFV B175L. Consequently, this interaction inhibits the interaction between cGAMP and STING, thereby inhibiting downstream signaling including STING polymerization and phosphorylation of TANK-binding kinase 1 and interferon regulatory factor 3 for antiviral signaling. Taken together, these results highlight the critical role of ASFV B175L in suppression of IFN responses and provide a new target to guide live-attenuated ASFV vaccine.

**IMPORTANCE:**

African swine fever virus (ASFV), the only known DNA arbovirus, is the causative agent of African swine fever (ASF), an acutely contagious disease in pigs. ASF has recently become a crisis in the pig industry in recent years, but there are no commercially available vaccines. Studying the immune evasion mechanisms of ASFV proteins is important for the understanding the pathogenesis of ASFV and essential information for the development of an effective live-attenuated ASFV vaccines. Here, we identified ASFV B175L, previously uncharacterized proteins that inhibit type I interferon signaling by targeting STING and 2′3′-cGAMP. The conserved B175L-zf-FCS motif specifically interacted with both cGAMP and the R238 and Y240 amino acids of STING. Consequently, this interaction interferes with the interaction of cGAMP and STING, thereby inhibiting downstream signaling of IFN-mediated antiviral responses. This novel mechanism of B175L opens a new avenue as one of the ASFV virulent genes that can contribute to the advancement of ASFV live-attenuated vaccines.

## INTRODUCTION

The cytosolic enzyme known as cyclic GMP-AMP synthase (cGAS) is activated by microbial double-stranded DNA (dsDNA) and serves as a pattern recognition receptor (PRR). It catalyzes the production of a unique intracellular second messenger called 2′3′-cGAMP (cGAMP), which belongs to the class of cyclic dinucleotides (CDNs). This cGAMP molecule, in turn, activates the stimulator of interferon genes protein (STING) (also named TMEM173, ERIS, MITA, or MPYS) (1). STING is an evolutionarily conserved, endoplasmic reticulum (ER) resident, ~40 kDa dimeric transmembrane adaptor for CDNs that perform multiple functions during infections, autoimmune diseases, and cancers (2). cGAMP-bound STING translocates from the ER to Golgi via the ER-Golgi intermediate compartment (ERGIC), in which STING recruits TANK-binding kinase 1 (TBK1); the latter then phosphorylates interferon regulatory factor 3 (IRF3). Phosphorylated IRF3 dimerizes and translocates to the nucleus to trigger expression of type I interferon (IFN-I) (3). STING translocation to the ERGIC triggers lipidation of microtubule-associated protein 1 A/1B-light chain-3, thereby inducing autophagy to clear pathogens from the cytosol; this process is a primordial function of the cGAS-STING axis that is independent of IFN-I. The result is activation of nuclear factor κB (NF-κB) via TBK1 and inhibitor-κB kinase ε (IKKϵ), which, in turn, orchestrates cellular resistance to invading pathogens (4, 5). Therefore, STING is a master molecule that determines the fate of microbial pathogens by sensing “foreign” DNA during infections.

African swine fever (ASF) is a notifiable, transboundary animal disease that is lethal to domestic pigs and wild boars. It is a highly contagious hemorrhagic fever for which there is no commercially available vaccine, and its unprecedented spread across Europe, Africa, and Asia is threatening the global pig industry. The ASF virus (ASFV) is a large arbovirus belonging to the family *Asfarviridae* (6). It is an icosahedral DNA virus measuring 200 nM in diameter and comprises an envelope, a capsid, an inner capsule membrane, a core shell, and an inner core. The viral genome is a linear dsDNA (170–190 kb long) molecule with covalently closed ends. The genome encodes 150–200 viral proteins, including 68 structural proteins and more than 100 non-structural proteins (7). ASFV targets the swine monocyte/macrophage lineage for replication (8). ASFV infection triggers a battle between the virus and the host’s innate antiviral immune responses. Viral proteins involved in immunoregulation play a significant role in virus replication (9). In recent studies, it has been demonstrated that ASFV utilizes multiple mechanisms to evade host defense systems. For instance, M1249L inhibits interferons (10), S273R affects inflammatory responses (11), A224L influences apoptosis (12), and EP402R impacts adaptive immunity (13).

Here, we show for the first time that ASFV B175L acts as a specific negative regulator of IFN-I signaling by targeting STING and cGAMP through its conserved MYM-type Zinc finger with FCS sequence motif (zf-FCS motif). The findings reveal a novel mechanism of immune evasion that could facilitate the development of new and effective live-attenuated vaccines against ASF.

## RESULTS

### ASFV B175L inhibits STING-mediated activation of the IFN-β promoter

To identify specifically how ASFV genes modulate the host defense mechanisms through STING, we used a dual-luciferase reporter assay to screen our ASFV gene library containing 60 Flag-tagged ASFV genes. For this purpose, plasmids carrying STING-Flag, TK *Renilla* luciferase, the IFN-β firefly luciferase promoter, and relevant ASFV genes were transfected into HEK293T cells, and STING-mediated IFN-β promoter activity was assessed. As shown in Fig. S1A, we identified seven ASFV genes that inhibited STING-mediated IFN-β luciferase induction markedly; among these, B175L was the most potent. These preliminary results suggest that ASFV B175L could be a novel ASFV virulence gene whose contribution to immune evasion is likely to occur via STING. B175L has been predicted to be a late viral gene when compared to Vaccinia virus (VACV) A1 and A2 sequences (14), yet no experimental evidence has been found to date. To comprehend the stage at which B175L is expressed, we conducted Quantitative Reverse Transcription PCR (qRT-PCR) analysis on ASFV-infected primary porcine alveolar macrophage (primary PAM) cells. As shown in Fig. S1B, our findings indicate that B175L is expressed at early time points and its expression gradually increases over time. This pattern is similar to the observed behavior of ASFV B646L (a late viral protein). These results indicate that ASFV B175L may contribute to ASFV pathogenesis not only in late stages but also at early stages.

### B175L negatively regulates antiviral immune responses

**Fig 1:**
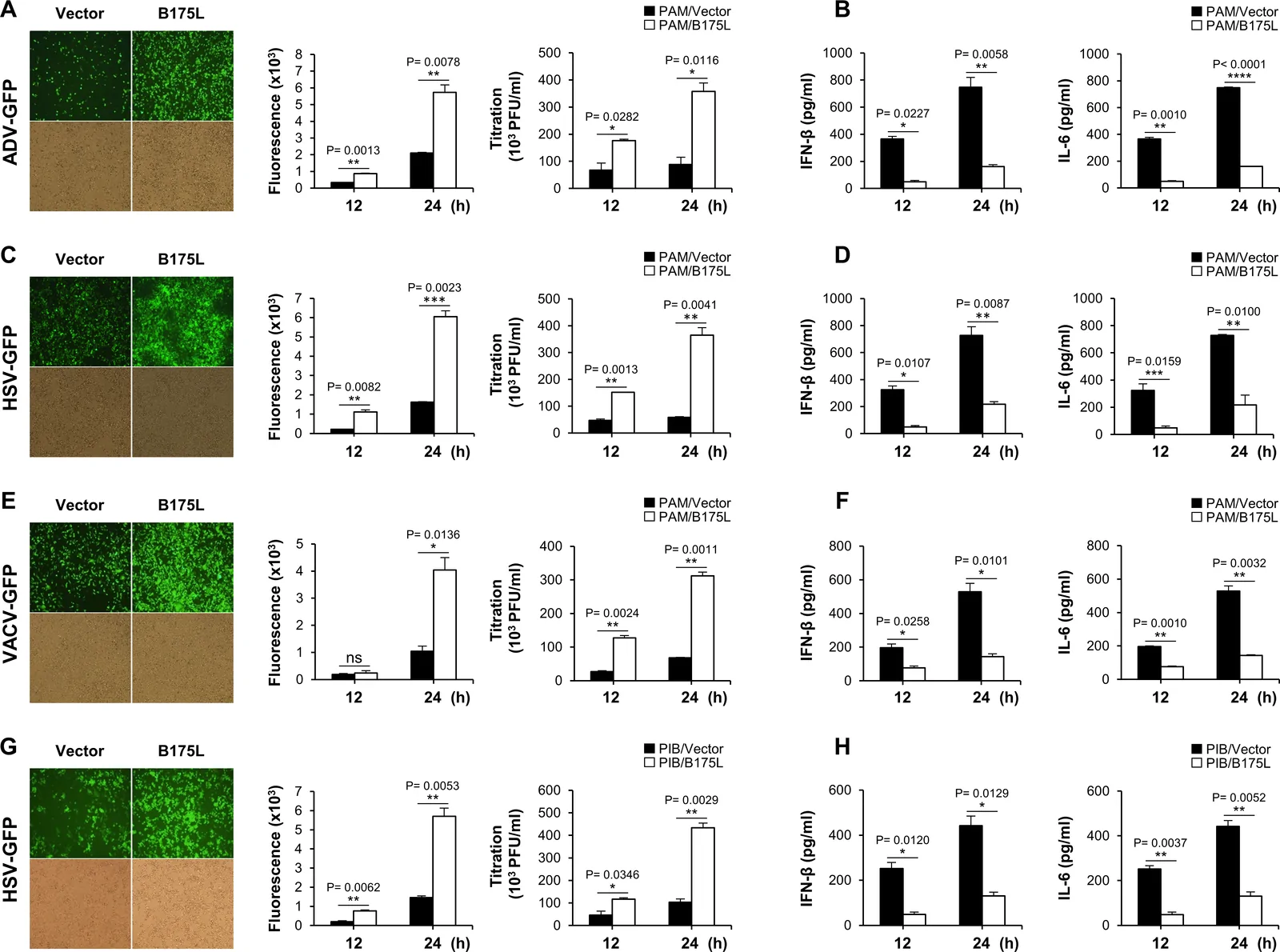
B175L negatively regulates antiviral immune responses in stable PAM cells. PAM cells stably expressing B175L-Flag or a control plasmid were infected with ADV-GFP, HSV-GFP, or VACV-GFP (multiplicity of infection (MOI) = 1.0). The GFP images were captured at 24 hpi using fluorescence microscopy and quantified at 12 and 24 hpi using the fluorescence modulator. Virus titers of each sample were determined by standard plaque assay in A549 and Vero cells (A, C, and E). Porcine IFN-β and IL-6 concentrations in the cell culture supernatant collected at 12 hpi and 24 hpi were estimated by enzyme-linked immunosorbent assay (ELISA) (B, D, and F). (G and H) GFP images, fluorescence level, virus titer, IFN-β, and IL-6 ELISA from HSV-GFP-infected stable PIB cells. The data represent at least two independent experiments with similar results, and the values are expressed as means and SD for three biological replicates. Student’s *t*-test: *P  <  0.05; **P  <  0.01; ***P  <  0.001; ****P  <  0.0001; ns, not significant.

**Fig 2:**
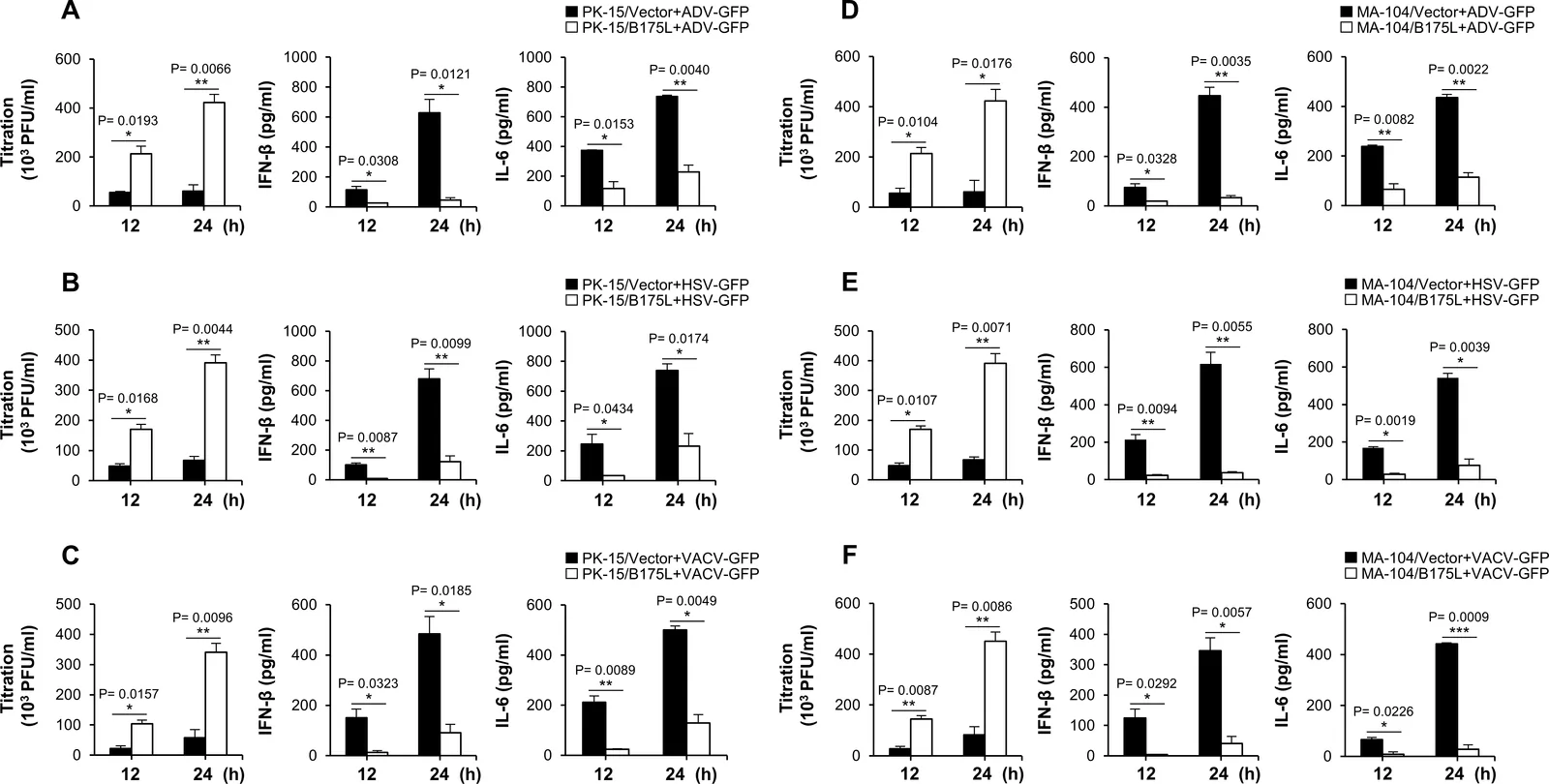
B175L downregulates host immune responses in PK-15 and stable MA-104 cells. PK-15 cells transiently expressing B175L-Flag or vector plasmid (**A–C**), as well as stable MA-104 cells (**D–F**), were infected with ADV-GFP, HSV-GFP, or VACV-GFP (MOI = 1.0). The virus titers of each harvested sample at the indicated time points were determined by the standard plaque assay in A549 and Vero cells. Concentrations of porcine IFN-β and IL-6 in the cell culture supernatant collected at 12 hpi and 24 hpi were estimated using ELISA. The data represent at least two independent experiments with similar results, and the values are expressed as means and SD for three biological replicates. Student’s *t*-test: *P  <  0.05; **P  <  0.01; ***P  <  0.001; ****P  <  0.0001; ns, not significant.

To determine whether B175L plays an antiviral role in response to DNA virus infection *in vitro*, we established stable expressions of B175L-Flag and control plasmids in porcine alveolar macrophages (PAM) and porcine-immortalized, bone marrow-derived macrophage (PIB) cells (Fig. S2A and B). The PAM cells stably expressing B175L and infected with adenovirus-green fluorescent protein (GFP) (ADV-GFP), herpes simplex virus-GFP (HSV-GFP), or VACV-GFP viruses, along with stable PIB cells infected with HSV-GFP, displayed heightened levels of fluorescence and increased virus titers (Fig. 1A, C, E, and G). Additionally, these cells displayed reduced expression of IFN-β and interleukin 6 (IL-6) compared to the control cells (Fig. 1B, D, F, and H). Furthermore, we introduced transient expressions of B175L-Flag and control plasmids into porcine kidney epithelial cells (PK-15) and established stable expressions in monkey kidney epithelial (MA-104) cells (Fig. S2C and D), followed by infection with the aforementioned viruses. As anticipated, B175L expression in both cell lines led to higher GFP expression and increased fluorescence intensity compared to the control (Fig. S2E and F). To validate the results, we measured virus replication, IFN-β, and IL-6 levels. As illustrated in Fig. 2A-C, we observed elevated virus titers in B175L-transfected PK-15 cells. Measurement of IFN-β and IL-6 levels after virus infection revealed lower levels in B175L-transfected cells than in control cells. Similar results were also obtained for infected stable MA-104 cells (Fig. 2D-F). Collectively, these findings suggest that B175L inhibits antiviral responses in different host cells.

### B175L inhibits IFN-I signaling and transcription of IFN-related genes

**Fig 3:**
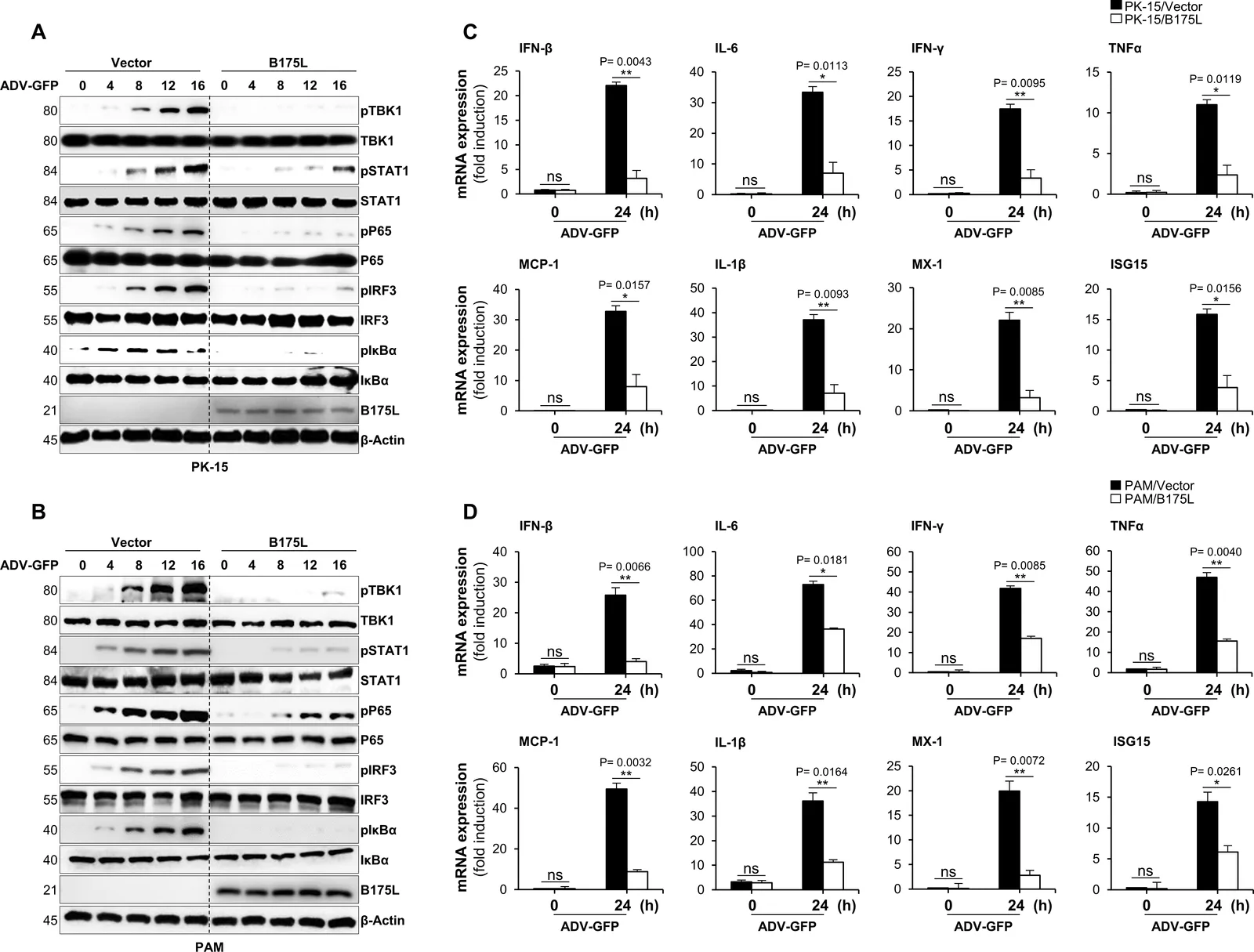
B175L inhibits cGAS-STING pathway signaling and the transcription of antiviral genes. (**A and B**) B175L-expressed PK-15 or stable PAM cells and control cells were infected with ADV-GFP (MOI = 1.0) and harvested at the indicated time points. The phosphorylated vs intact forms of TBK1, STAT1, p65, IRF3, IκBα, and the expression of B175L-Flag were detected by immunoblotting. The β-actin was used as the internal control for equal protein amounts in samples. Protein sizes are expressed in kilodaltons (kDa). All the immunoblot data are representative of at least two independent experiments, each with similar results. (**C and D**) PK-15 or stable PAM cells harboring B175L-Flag and control cells were infected with ADV-GFP (MOI = 1.0) and harvested at the indicated time points. The total RNA was extracted from cells, and the transcription of antiviral genes was analyzed by qRT-PCR. The mRNA induction levels in 0 h and 24 h vector- or B175L-expressing cells were compared. The mRNA expression levels were analyzed according to the delta–delta CT (2^−ΔΔCT^) method, and β-actin or glyceraldehyde-3-phosphate dehydrogenase was used as an internal housekeeping gene for normalization. The data represent at least two independent experiments with similar results, and the values are expressed as means and SD for three biological replicates. Student’s *t*-test: *P  <  0.05; **P  <  0.01; ***P  <  0.001; ****P  <  0.0001; ns, not significant.

To further examine the effects of B175L on virus-mediated activation of the IFN-I signaling cascade, we analyzed virus-induced phosphorylation of TBK1, signal transducer and activator of transcription 1 (STAT1), IRF3, p65 (RelA), and inhibitor of nuclear factor kappa B (IκBα), all of which are key signaling molecules within the IFN-I and NF-κB pathways. B175L-overexpressing PK-15, stable PAM, and the corresponding control cells were infected with ADV-GFP and harvested 0, 4, 8, 12, and 16 h later for immunoblot analysis. The B175L-expressed PK-15 and stable PAM cells showed a marked decrease in phosphorylation of TBK1, STAT1, IRF3, p65, and IκBα compared with control cells (Fig. 3A and B). Next, we measured expression of mRNA encoding IFN-related genes, such as IFN-β, IL-6, IFN-γ, TNFα, MX-1, MCP1, ISG15, and IL-1β using real-time PCR. B175L-overexpressing PK-15, stable PAM, and control cells were harvested at the indicated times post-infection with ADV-GFP, and total RNA was extracted. Compared with control cells, B175L-overexpressing PK-15 and stable PAM cells showed lower expression of mRNA encoding IFN-β and other IFN-related genes (Fig. 3C and D). These results suggest that B175L is a negative regulator of IFN-I signaling that is induced by virus infection.

### B175L directly interacts with STING

**Fig 4:**
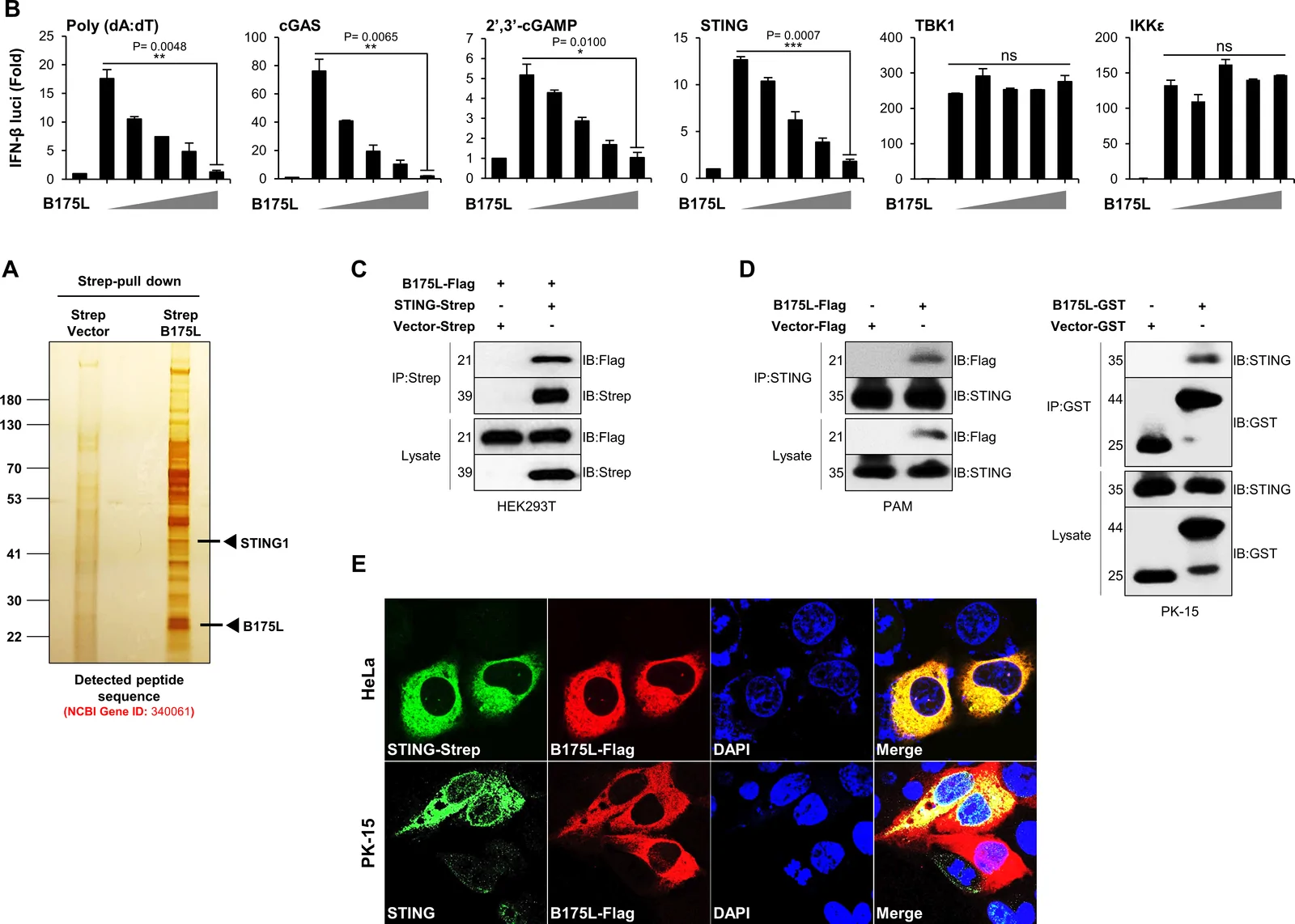
B175L interacts with STING. (**A**) Silver staining for the B175L interactome assay followed by mass spectrometry. The arrow indicates the protein expression of B175L-Strep and the expected protein expression of STING (UniProt: Q86WV6). (**B**) HEK293T cells were co-expressed with IFN-β promoter (firefly), TK-*Renilla* (internal control), stimulators [Poly (dA:dT), or STING, or TBK1, or IKKε], and increasing doses (50, 100, 200, 400 ng) of B175L-Flag for 24 h. The luciferase activity of each sample was quantified with a dual-luciferase reporter assay system. To measure cGAS and cGAMP-induced luciferase activities, 93-Dual hSTING-A162 cells were stimulated with 3× Flag cGAS or 4 µg/mL of cGAMP for 12 h. The IFN-β-dependent expression of Lucia luciferase was estimated by QUANTI-Luc. The data represent at least three independent experiments with similar results, and the values are expressed as means and SD for three biological replicates. Student’s *t*-test: *P < 0.05; **P < 0.01; ***P < 0.001; ****P < 0.0001; ns, not significant. (**C**) HEK293T cells transfected with B175L-Flag and STING-Strep plasmids were subjected to immunoprecipitation with an anti-Strep antibody followed by immunoblotting with the indicated antibodies. (**D**) PK-15 cells transfected with B175L-Flag and control plasmids or stable PAM cells harboring B175L-Flag or control plasmids were stimulated with HSV-GFP (MOI = 1.0). After 24 hpi, the cells were harvested and subjected to immunoprecipitation and immunoblotting. Protein sizes are expressed in kilodaltons (kDa). All the immunoblot data are representative of at least two independent experiments, each with similar results. (**E**) Confocal microscopy assays were used to examine the co-localization of B175L and STING in PK-15 cells upon VACV wild-type (MOI = 1.0) stimulation and in HeLa cells. The nuclei were stained with DAPI (blue). The arrow indicates the co-localized B175L and STING proteins at the endogenous level.

To further determine the specific target molecule of B175L during immune evasion, we expressed Strep-tagged B175L in HEK293T cells and conducted a large-scale Strep pull-down assay. Surprisingly, mass spectrometry analysis of the products of the Strep pull-down assay identified STING (UniProt: Q86WV6) as a candidate target of B175L (Fig. 4A). We performed the dual-luciferase reporter experiment on HEK293T cells to better understand the impact of B175L on immune evasion. B175L-overexpressing HEK293T cells suppressed Poly (dA:dT)-, cGAS-, and STING-mediated IFN-β promoter activation in a dose-dependent manner, as well as activation of 2′3′-cGAMP-induced IFN-β promoter in 293-Dual hSTING-A162 cells. However, there was no detectable fold change in TBK1- or IKKε-mediated IFN-β promoter activation, despite increasing doses of B175L (Fig. 4B). This suggests that ASFV B175L acts immediately upstream of TBK1. To confirm the mass spectrometry results, we performed immunoprecipitation assays with B175L-Flag and STING-Strep plasmids. As expected, immunoblot analysis revealed direct interaction between B175L and STING in HEK293T cells (Fig. 4C). Next, we investigated this interaction at the endogenous level. Stable PAM cells or B175L-transfected PK-15 were infected with HSV-GFP (MOI = 1.0), followed by immunoprecipitation. The results demonstrate that the B175L and STING interaction occurred at the endogenous level in stable PAM and PK-15 cells (Fig. 4D). Finally, as shown in Fig. 4E, a confocal microscopy assay confirmed overexpression and endogenous co-localization of B175L and STING in HeLa and PK-15 cells. Taken together, these results suggest that ASFV B175L triggers a novel mechanism that antagonizes the IFN-I pathway targeting STING.

### The B175L zf-FCS motif inhibits the interaction between STING and cGAMP

**Fig 5:**
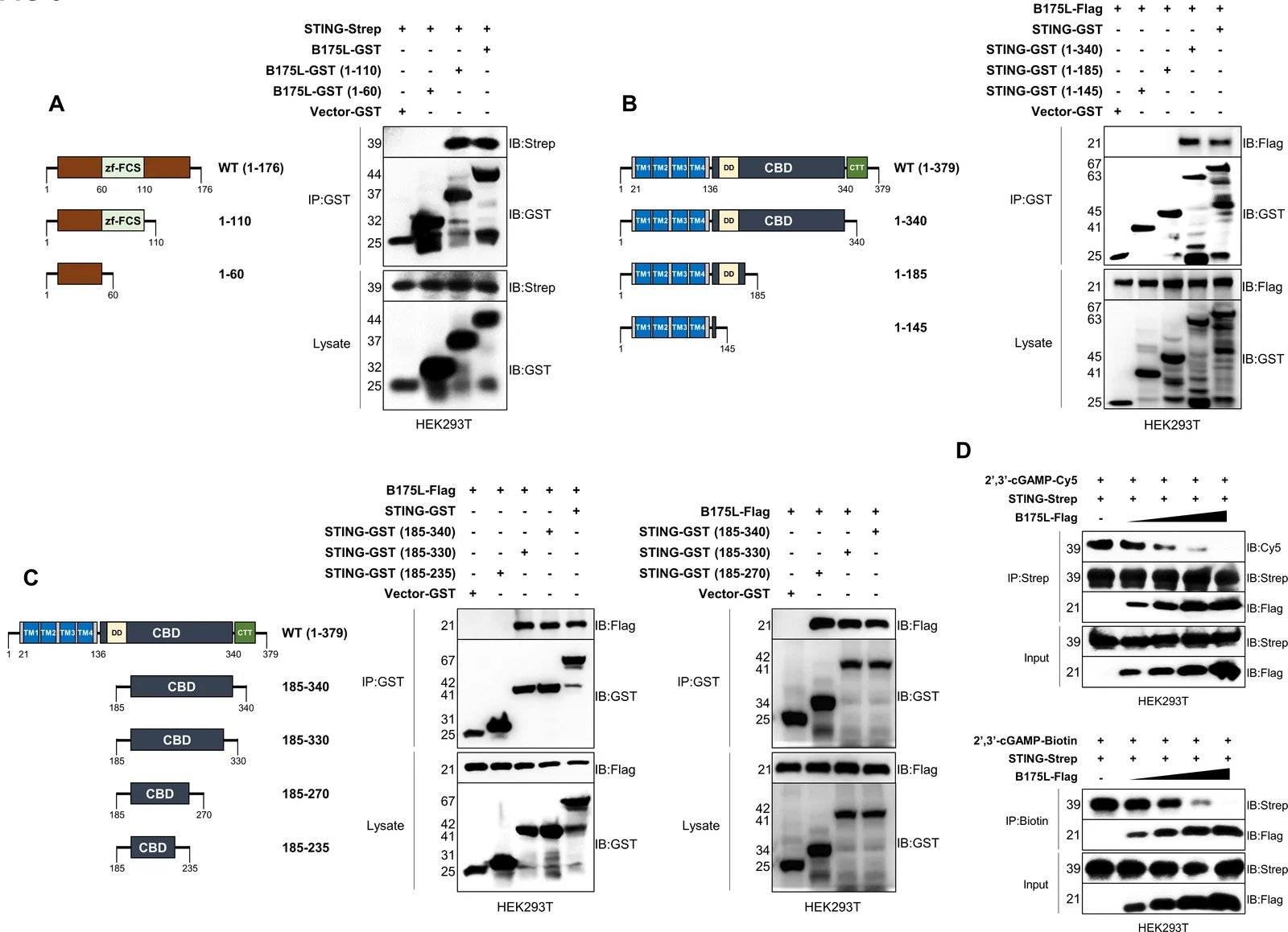
B175L inhibits STING and cGAMP interaction. (**A**) GST-tagged B175L domains (aa 1–60 and aa 1–110) with or without the zf-FCS motif (left). Immunoprecipitation of GST-tagged B175L and its domain constructs with STING-Strep (right). Protein expressions were determined by anti-GST and anti-Strep antibodies. (**B and C**) Domain analysis to find the interface of STING that interacts with the zf-FCS motif of B175L-Flag. Control plasmid, GST-tagged STING (wild type), and its constructs (aa 1–145, aa 1–185, aa 1–340, aa 185–235, aa 185–270, aa 185–330, and aa 185–340) were transfected into HEK293T cells and subjected to GST immunoprecipitation followed by immunoblotting with anti-Flag and anti-GST antibodies. (**D**) *In vitro* competition assay with cGAMP, STING, and B175L. 2′3′-cGAMP-Cy5 conjugate or 2′3′-cGAMP-Biotin conjugate was incubated with affinity-purified STING and B175L (1, 3, 6, and 10 µg). After the incubation, the reaction mixture was pulled down with Strep beads or Dynabeads M-280 Streptavidin and subjected to immunoblotting analysis. Protein sizes are expressed in kilodaltons (kDa). All the immunoblot data are representative of at least two independent experiments, each with similar results.

Next, we identified the domain within B175L that interacts with STING. B175L contains an MYM-type zinc finger (ZF) domain with an FCS sequence motif. MYM-type ZFs work as transcriptional trans-activators of late vaccinia viral genes, and orthologs exist in all nucleocytoplasmic large DNA viruses (15). Here, we constructed two GST-tagged fragments of B175L (aa 1–60 and aa 1–110) and immunoprecipitated them with STING. Interestingly, we found that B175L interacted with STING through its conserved zf-FCS motif (Fig. 5A). Accordingly, we tested which domain of STING is essential for binding to the zf-FCS motif of B175L. STING is an ER resident protein that retains four transmembrane (TM) helices, which form the TM domain (TMD), followed by the cyclic dinucleotide binding domain (CBD) that binds to cGAMP. The STING C-terminal tail recruits TBK1 and IRF3 to potentiate expression of IFN-I during virus infection (16). Recent studies identified the essential amino acids in STING. Substituting several residues within human STING (i.e., I10Q, R14A, E68A, E69A, R238A, Y240A, N242A, E260A, Q273A, A277Q, L333A, R334A, S366A, and L374A) abolished cGAMP-induced STING signaling (17). First, we constructed three GST-tagged STING domains (aa 1–145, aa 1–185, and aa 1–340) and performed immunoprecipitation assays with B175L. Our results demonstrated that the zf-FCS motif of B175L interacted with STING at its CBD, which contains amino acid residues essential for cGAMP binding, STING polymerization, and autophagy (Fig. 5B). To further examine the specific binding site, we made four additional domain constructs (aa 185–235, aa 185–270, aa 185–330, and aa 185–340) and conducted binding assays. We found that B175L and STING interaction was mediated by aa 185–330 of STING, more specifically, aa 185–270, which includes known cGAMP binding amino acids R238, Y240, N242, and E260 (Fig. 5C). Thus, we identified the specific domains of B175L and STING that are responsible for the interaction.

Next, we conducted *in vitro* competition assays involving cGAMP, STING, and B175L. To achieve this, we eluted the relevant proteins from HEK293T cells and utilized them for *in vitro* competition assays, introducing increasing concentrations of B175L. As expected, we observed that the affinity of cGAMP-Cy5 for STING decreased in the presence of increasing doses of B175L (Fig. 5D, upper panel). Additionally, we obtained similar consistent results for the 2′3′-cGAMP-Biotin conjugate (Fig. 5D, lower panel). These findings illustrate that pB175L disrupts the interaction between cGAMP and STING in a dose-dependent manner.

### Mutations at R238 and Y240 of STING abolish its interaction with B175L

**Fig 6:**
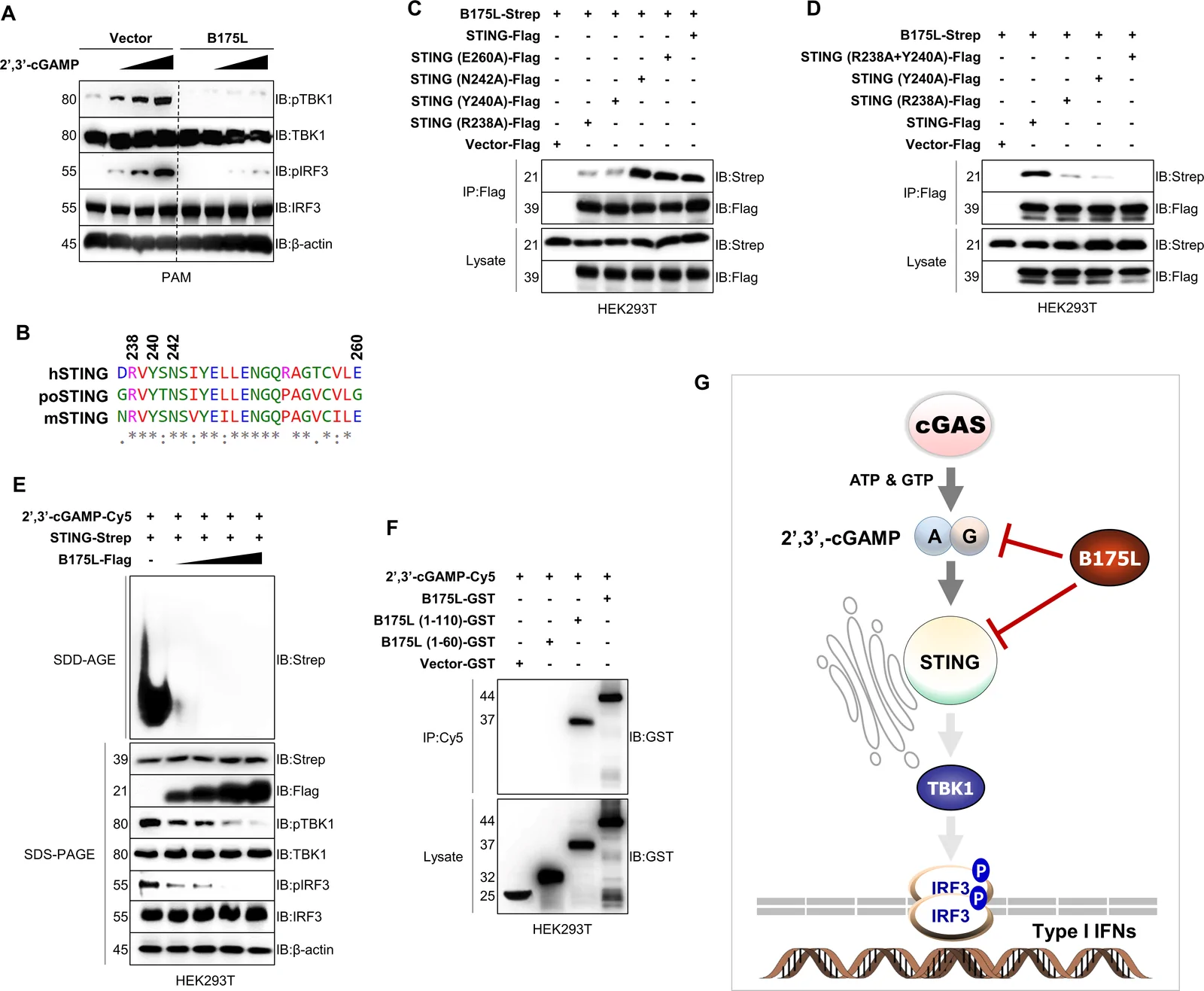
B175L interaction requires R238A and Y240A of STING. (**A**) PAM cells that stably express B175L inhibit cGAMP-induced phosphorylation of TBK1 and IRF3. Stable PAM cells were stimulated with 1, 3, and 5 µM of 2′3′-cGAMP. After 12 h post-transfection, the phosphorylated vs nonphosphorylated TBK1 and IRF3 and the expression of B175L-Flag were determined by immunoblotting. (**B**) The sequence alignment shows well-studied cGAMP-specific binding amino acids of STING R238, Y240, and N242 that share a conserved interface among human (hSTING), porcine (poSTING), and mouse (mSTING). (**C and D**) STING R238A and Y240 abolished B175L and STING binding. Expression plasmids encoding B175L, full-length STING, and its mutants were transfected into HEK293T cells, followed by immunoprecipitation and immunoblotting. (**E**) B175L inhibited STING polymerization in a dose-dependent manner. HEK293 cells transfected with STING-Flag expression plasmids and increased doses of B175L-Strep plasmids were stimulated with 4 µM of cGAMP. After 4 h, cell lysates were resolved by Semi-denaturating detergent agarose gel electrophoresis (SDD-AGE) (top) or SDS-PAGE (bottom) with the indicated antibodies. (**F**) cGAMP interacts with B175L. 2′3′-cGAMP-Cy5 conjugate was incubated at 37°C with affinity-purified proteins of B175L-GST or its constructs. After 2 h, the reaction mixture was pulled down with an anti-Cy5 antibody and subjected to SDS-PAGE. Protein sizes are expressed in kilodaltons (kDa). All the immunoblot data are representative of at least two independent experiments, each with similar results. (**G**) Graphical summary of B175L immune evasion. ASFV B175L, a previously uncategorized protein, evades antiviral immune responses targeting the central immune molecule STING and its ligand cGAMP, which cause inhibition of downstream immune signaling via TBK1 and IRF3.

To gain a deeper insight into the B175L and STING binding interface, we transfected cGAMP into stable PAM cells and checked the phosphorylation levels of TBK1 and IRF3. As shown in Fig. 6A, cGAMP-stimulated phosphorylation of TBK1 and IRF3 in control cells increased in a dose-dependent manner. By contrast, cGAMP-induced downstream signaling was abolished in B175L stable PAM cells. Our sequence alignment shows that the cGAMP-specific binding residues within STING are conserved among species; the R238, Y240, and N242 sites are shared equally by human (hSTING), porcine (poSTING), and mouse STING (mSTING) (Fig. 6B). Since we already identified B175L and STING binding in stable PAM cells and PK-15 cells (Fig. 4D), we hypothesized that conserved residues R238, Y240, and N242 of STING could be relevant binding sites for B175L. To explore this possibility, we constructed Flag-tagged R238A, Y240A, N242A, and E260A (another known residue) mutants and conducted immunoprecipitation experiments with B175L. We observed that mutations at N242 and E260 did not impact STING-B175L binding. However, the R238A and Y240A mutations each reduced the binding affinity (Fig. 6C), and the R238A + Y240A mutant completely abolished binding of B175L to STING (Fig. 6D), suggesting that the zf-FCS motif of B175L interacts with R238 and Y240 of STING, thereby masking the cGAS-induced cGAMP-STING interaction. Recent data show that binding of cGAMP to STING generates conformational changes at the ER, resulting in polymer formation before STING traffics to the Golgi, where downstream signaling molecules are recruited. This ER-to-Golgi migration occurs via coat protein complex-II vesicles, and the process is independent of STING C-terminal tail (CTT) and palmitoylation at the Golgi (18).

Therefore, we performed a STING aggregation assay to examine STING polymerization in the presence of B175L. As predicted, B175L inhibited cGAMP-induced polymerization of STING in a dose-dependent manner (Fig. 6E). Since both cGAMP and B175L target R238 and Y240 of STING, we argued that B175L also binds to cGAMP. To check this, we performed an *in vitro* binding assay with 2′3′-cGAMP-Cy5 and B175L. Interestingly, we found that B175L interacted with 2′3′-cGAMP-Cy5, specifically through the zf-FCS motif, which is important for B175L and STING interaction (Fig. 6F). Taken together, these data suggest that ASFV B175L, a previously uncategorized protein, enables the virus to evade antiviral immune responses by targeting the central immune molecule STING and its ligand cGAMP, thereby inhibiting downstream immune signaling via TBK1 and IRF3 (Fig. 6G).

## DISCUSSION

The innate immune system uses multiple germ-line encoded PRRs to recognize various pathogen-associated molecular patterns such as viral DNA to orchestrate antiviral immune responses. There are many different PRRs that can detect viral DNA in the cytosol (19, 20). One example is the cGAS-STING-IRF3 axis, in which binding of cytosolic viral DNA to cGAS triggers its catalytic activity to generate cGAMP, which then interacts with the ER resident protein STING. The cGAMP and STING complex undergoes structural changes and traffics to the Golgi via ERGIC, where it recruits TBK1 and IRF3 prior to activating IFN-I (21). The STING protein comprises three specific domains: a TMD, a CBD, and a CTT. The CBD and CTT domains form a butterfly-shaped domain-swapped homodimer under steady-state conditions. After binding to cGAMP, the STING homodimer undergoes extensive conformational changes, referred to as STING activation. During activation, STING releases its hidden CTT and undergoes polymerization and ubiquitination (5). Activated STING then transverses to the Golgi apparatus, where it is palmitoylated to induce STING-dependent downstream signaling (22). Translocated STING recruits TBK1 to its CTT through a PXPLRXD (where X refers to any residue) motif, and the TBK1 and STING complex phosphorylates IRF3, which moves into the nucleus as a dimer to induce secretion of IFN-I (23).

Viruses have evolved specific strategies to escape host immune responses. Numerous viral proteins that interfere with STING activation and trafficking. For example, protein kinases (US3, VP24), protein-protein interaction inhibitors, deubiquitinase UL36USP, and viral ubiquitin ligase ICP0 (encoded by HSV-1) antagonize STING signaling (24). Human papillomavirus E7 and ADV E1A obstruct STING via viral LXCXE motifs (25). Hepatitis C virus NS4B and dengue virus NS2B3 proteins cleave STING (26), whereas the HSV-1 VP24 and Kaposi’s sarcoma-associated herpesvirus vIRF1 block the STING-TBK1 interaction. HSV-1 VP1-2 and human T-lymphotropic virus type 1 Tax deubiquitinate STING, HSV-1 γ134.5 abrogates STING trafficking to the Golgi, and the UL82 of human cytomegalovirus iRhom2 mediates STING-TRAPβ complex assembly to interrupt TBK1 and IRF3 recruitment (27). ASFV is a large, double-stranded DNA virus that infects domestic or feral swine of all ages causing 100% mortality. A more in-depth understanding of ASFV-host interactions requires high-quality, full-length genomic sequences of a variety of ASFV genotypes. However, most ASFV open reading frames are only predictions, and their functions are still experimentally unknown (28).

ASFV modulates the IFN and NF-κB pathways for efficient replication. Recent studies show that ASFV virulent strains Armenia/07, 22653/14, L60, and low virulent ASFV/NHV suppress IFN and IFN-stimulated genes in infected cells (29 – 31). Among the many ASFV-encoded proteins, MGF360-15R, I329L, E120R, I215L, A137R, MGF360-11L, and M1249L suppress IFN-I (32 – 37). MGF360-12L and DP96R inhibit the IFN-I and NF-κB pathways (38, 39). Yang et al. (40) reported the negative regulation of the cGAS-STING pathway by ASFV MGF505-11R. They found that MGF505-11R interacted with STING and increased its degradation through the lysosomal, ubiquitin-proteasome, and autophagy pathways (40). Another study revealed the role of MGF505-7R against STING-dependent antiviral responses. MGF505-7R bound to STING and inhibited the cGAS-STING pathway, while upregulating expression of ULK1 to degrade STING via autophagy (41). Zheng et al. reported the negative effects of ASFV p17 protein on STING signaling. They found that binding of p17 to STING interfered with recruitment of TBK1 and IKKε (42). We recently discovered a novel immune evasion mechanism mediated by ASFV EP364R and C129R, which share nuclease homology, and block cGAMP via phosphodiesterase activity (43).

In this study, we identified a novel molecular anti-immune mechanism mediated by B175L, an uncategorized protein of ASFV. First, we found that B175L impairs immunity in transfected cells. Expression of B175L in PAM, PK-15, PIB, and MA-104 cells increased replication of GFP-tagged viruses. In addition, cytokine levels, antiviral signaling, and transcription of IFN-related genes fell. Second, pull-down assays followed by a mass spectrometry analysis identified STING (UniProt: Q86WV6) as a binding partner for B175L. HEK293T cells, which are used primarily in cellular biology experiments, are derived from HEK293 cells with stable expression of SV40 polyomavirus large T antigen (LT), which enhances replication of plasmid DNA to achieve a high copy number (44). HEK293T cells are deficient in both cGAS and STING (45). Previously, it was thought that lack of STING expression by HEK293T cells was caused by SV40 LT. However, a recent finding revealed that the SV40 LT is not responsible for loss of STING from HEK293T cells (46). The authors of that study suggest that this cell-specific difference in terms of STING expression could be caused by differentially expressed genes (DEGs) or epigenetic reprogramming. Thus, the most concise explanation for STING detection upon silver staining of B175L-transfected HEK293T cells is alteration of DEGs by B175L through an unknown mechanism.

Our transient and endogenous immunoprecipitation assays confirmed the *in vitro* B175L and STING interaction which was validated by confocal microscopy analysis. Third, we identified the regions of B175L and STING that are required for this interaction. Analysis of the B175L domain revealed that the zf-FCS motif of B175L is crucial. ZF proteins are one of the most abundant groups of proteins and fulfill a wide range of molecular functions. zf-FCS was first identified as an MYM family protein related to myeloproliferative syndrome and mental retardation. These proteins are present in viruses, eubacteria, archaea, metazoa, and plants. Previous reports show that zf-FCS accommodates nucleic-protein and protein-protein interactions (47). Analysis of the STING domain revealed that the STING CBD (aa 185–270) harbors four cGAMP binding residues (R238, Y240, N242, and E260) (17), suggesting that B175L disrupts cGAMP and STING binding. Furthermore, our experiments using cGAMP conjugates showed that B175L competes with cGAMP for binding to STING.

Finally, to get a deeper insight into the B175L and STING interaction, we transfected cGAMP dose dependently into stable PAM cells and examined phosphorylation of TBK1 and IRF3. As expected, we observed almost zero phosphorylation of TBK1 and IRF3 in B175L stable PAM cells, despite the increasing amounts of cGAMP (Fig. 6A). Mutation analysis revealed that STING R238 and Y240 are essential for the B175L and STING interaction, while N242 and Y240 are not. This indicates that STING R238 and Y240 are key locations for cGAMP-favored activation of STING signaling. Therefore, the R238A and Y240A mutants (in which the strong affinity of cGAMP for STING is abolished) can be useful as a control when studying cGAMP and STING interactions (17). Our sequence analysis revealed that R238 and Y240 are conserved among species. Furthermore, we found that the zf-FCS motif of B175L not only binds to STING but also interacts directly with cGAMP. Overall, binding of B175L to STING and cGAMP abrogates the STING polymerization, which could disrupt ERGIC/Golgi trafficking.

In conclusion, we show here that the previously uncategorized ASFV protein B175L is an antagonist of type-I IFN. B175L interacts with cGAMP and STING to impair STING-mediated transduction of antiviral signals. Taken together, these data increase our understanding of the diverse mechanisms underlying ASFV pathogenesis and open up new avenues of research aimed at virus attenuation and future ASFV vaccine development.

## MATERIALS AND METHODS

### Chemicals and antibodies

2′3′-cGAMP-Biotin conjugate (AAT Bioquest; 20316), 2′3′-cGAMP-Cy5 (AAT Bioquest; 20318), GlutaMAX Supplement (Gibco), Trypsin-EDTA (Gibco) Normocin-Antimicrobial Reagent (Invivogen), Blasticidin (Invivogen), Hygromycin B Gold (Invivogen), Zeocin (Invivogen), Puromycin (Invivogen), Poly(dA:dT) (Invitrogen), Lipofectamine 2000 (Invitrogen), Polyethyleneimine/PEI (Polysciences; 9002-98-6/26913-06-4), Protein A/G PLUS-Agarose (Santa Cruz Biotechnology; sc-2003), Halt Protease Inhibitor Cocktail (Thermo Scientific; 78429), Sepharose 6B (GE Healthcare; 17011001), Glutathione-conjugated Sepharose 4B (GST) beads (Cytiva), Strep-Tactin Sepharose resin (IBA Lifesciences; 2–1201-002), Quanti-Luc (Invivogen) were obtained commercially. The list of antibodies purchased from Cell Signaling Technology for this study included STING (D2P2F; 13647), TBK1/NAK (D1B4; 3504), STAT1 (42H3; 9175), NF-κB p65(D14E12; 8242), IRF3 (D83B9; 4302), IκBα (9242), pTBK1/NAK (D52C2; 5483), pSTAT1 (58D6; 9167), Phospho-NF-κB p65 (93H1; 3033), pIRF3 (4D4G; 4947), pIκBα (14D4; 2859), and Flag (M2) (8146). Other antibodies are as follows: Alexa Flour 488 (Abcam; 150077), Alexa Flour 647 (Abcam; 150079), StrepMAB-Classic HRP conjugate (IBA Lifesciences; 2-1509-001), Cy5 (Abcam: CY5-15), β-actin (Santa Cruz Biotechnology; sc-47778), and GST (Santa Cruz Biotechnology; sc-138).

### Cell culture and transfection

PK-15 cells (ATCC CCL-33), HeLa cells (ATCC CCL-2), HEK293T cells (ATCC CRL-11268), Vero cells (ATCC CCL-81), 293-Dual hSTING-A162 cells harboring the stable transfections of the A162 isoform of human STING (S162A) and are resistant to antibiotics such as blasticidin, hygromycin, and Zeocin (Invivogen), A549 cells (ATCC CCL-185), and MA-104 cells (CRL-2378.1) were cultured in Dulbecco’s Modified Eagle Medium (DMEM) (Cytiva), and PAM cells (ATCC CRL­2843) were cultured in RPMI Medium (Cytiva). Both mediums were supplemented with 10% fetal bovine serum (FBS) (Gibco) and 1% Anti-Anti (Gibco). PIB isolation was performed as described previously (48). PIB cells were cultured in RPMI Medium (Cytiva) supplemented with 10% FBS (Gibco), 1% Anti-Anti (Gibco), Normocin (100 µg/mL), Blasticidin (100 µg/mL), Hygromycin B Gold (50 µg/mL), and Zeocin (50 µg/mL). Primary PAM (Optipharm) were cultured in RPMI medium (Cytiva) supplemented with 10% FBS (Gibco) and 1% Penicillin-Streptomycin (Gibco). All the cell lines used in this study were maintained in a humidified 5% CO_2_ incubator at 37°C.

For plasmid transfection, PEI was used for HEK293T cells and Lipofectamine 2000 for all other cells, following the manufacturer’s instructions. Lipofectamine RNAiMAX was chosen for cGAMP transfection. PAM, MA-104, and PIB cells stably expressing the pIRES-Flag (control) or pIRES-B175L-Flag (B175L-Flag) were generated by supplementing the cell culture media with 2.0, 4.0, and 0.3 µg/mL of puromycin, respectively.

### Plasmids

Wild-type STING was amplified from template DNA using PCR and cloned into pIRES-Flag (STING-Flag), pEXPR-Strep (STING-Strep), and pEBG (STING-GST) vectors. The complete sequence of the ASFV B175L gene (FR682468.1: from 107540 to 108067) was cloned into pIRES-Flag (B175L-Flag), pEXPR-Strep (B175L-Strep), and pEBG vectors (B175L-GST); STING (STING 1–140, 1–185, 185–235, 185–270, 185–330, 185–340, and 1–340) and B175L (B175L 1–60 and 1–110) domain constructs were cloned to the pEBG vector. The cGAMP binding defective single-site (R238A, Y240A, N242A, and E260A) and the double-site (R238A + Y240A) STING mutants were generated using Mutation Generation System Kit (Thermo Scientific; F701) complying with the manufacturer’s instructions and cloned to pIRES-Flag vector. The PCR primers used for site-directed mutagenesis are listed in Table S1.

### Quantitative real-time PCR

B175L-Flag-transfected PK-15 or stably expressed PAM and control cells grown in 12-well tissue culture plates (3 × 10^6^ cells/well) were incubated in the humidified 5% CO_2_ at 37°C. After 12 h, cells were infected with ADV-GFP (MOI = 1.0), harvested at two time points (12 and 24 hpi), and stored at −80°C. Next, the total RNA was isolated using the RNeasy Micro Kit (Qiagen; 74004), and the complementary DNA (cDNA) was synthesized by reverse transcriptase (Toyobo; FSK-101). Finally, the qPCR was performed to quantify the different levels of cDNA using SYBR Green Q-PCR Master mix (SJ Bioscience; SG-SYBR-500) following the manufacturer’s protocol on a Rotor‐Gene Q (Qiagen). The gene-specific primer sets are listed in Table S2.

### Virus infection and replication assay

GFP-tagged viruses used in this study were ADV-GFP, HSV-GFP, and VACV-GFP. These viruses were amplified in PK-15 cells and titrated by plaque assay. The virus infection into cells was done in reduced serum (1% FBS)-containing medium for 2 h. The cell supernatants retaining uninfected viruses were replaced with a fresh culture medium afterward. The GFP images were captured by fluorescence microscope (200× magnification) at 24 hpi. The fluorescence intensity was measured from cell extracts harvested at 12 hpi and 24 hpi using the fluorescence modulator (GloMax-Multi Detection System, Promega). The cell extracts and supernatants received through the freeze-thawed process were used for the standard plaque assay in A459 (ADV-GFP) and Vero cells (HSV-GFP and VACV-GFP). For the ASFV infection experiment, primary PAM cells were infected with 1.0 MOI of ASFV (Korea/wildboar/Hwacheon/2020-2287). The cell pellets were harvested at 0, 3, 6, 9, 12, 15, and 18 hpi. Subsequently, RNA extraction was conducted, and qRT-PCR analysis was performed using B175L primers (Table S2). ASFV I73R (an early gene) and B646L (a late gene) were used as controls.

### Immunoblot analysis and immunoprecipitation

For immunoblot analysis, cells were harvested after 36 h post-transfection of relevant plasmids and lysed with radioimmunoprecipitation assay (RIPA) buffer [50 mM Tris–HCl, 150 mM NaCl, 0.5% sodium deoxycholate, 1% octylphenoxy poly(ethyleneoxy)ethanol, branched (IGEPAL)] containing phosphatase inhibitor (1 mM Na_3_PO_4_) and protease inhibitor cocktail. Then, the lysates were sonicated (30% amplitude, 10 s, three cycles for 500 µL of lysate) and centrifuged at 12,000 rpm at 4°C for 10 min. The received whole-cell lysates (WCLs) were 1:1 mixed with the sample buffer (Sigma; S3401), heated at 100°C for 10 min, and subjected to typical SDS-PAGE, followed by immunoblotting with the indicated antibodies. For immunoprecipitation, Sepharose 6B was added to WCLs (preclearance) and incubated in a rotter at 4°C for 3 h, followed by centrifugation at 12,000 rpm at 4°C for 3 min. Next, the WCLs were incubated with Strep-Tactin Sepharose resin, Glutathione-conjugated Sepharose 4B, or primary antibodies overnight at 4°C. Lastly, the immunoprecipitated beads were collected by centrifugation and washed with 300 mM NP40 lysis buffer before samples were prepared for SDS-PAGE. Concurrently, WCLs containing primary antibodies were incubated with Protein A/G PLUS‐Agarose for 4 h at 4°C. Afterward, the beads were prepared as above-mentioned. The next day, the protein-transferred polyvinylidene difluoride (PVDF) membranes were washed with Tris-buffered saline with Tween 20 (TBST) and incubated, adding the horseradish peroxidase-conjugated (HRP) mouse or rabbit secondary antibodies for 2 h at room temperature and washed again with TBST. All the PVDF membranes were visualized using an enhanced chemiluminescence detection system (ECL-GE Healthcare, Little Chalfont, United Kingdom) using a Las-3000 mini Lumino Image Analyzer.

### Semi-denaturating detergent agarose gel electrophoresis (SDD-AGE) assay

The SDD-AGE assay was done as previously described (49) with some modifications. In brief, HEK293T cells cultured in six-well plates were transfected with STING-Strep and B175L-Flag plasmids as indicated. On the following day, cells were stimulated with 4 µg/well of the cGAMP ligand for 4 h. Subsequently, the cells were rinsed with PBS, and the harvested cell extracts were lysed using RIPA buffer (50 mM Tris–HCl, 150 mM NaCl, 0.5% sodium deoxycholate, 1% IGEPAL), which contained 1 mM Na_3_PO_4_ and PI, for 4 h at 4°C on a rocker. A portion of the WCLs was used for SDS-PAGE, while the proteins eluted with glycine (immunoprecipitated proteins) were subjected to 1.5% SDD-AGE. For the SDD-AGE procedure, samples were loaded onto a 1.5% vertical agarose gel (with 1× Tris-acetate-EDTA (TAE) and 0.1% SDS) and underwent electrophoresis in the running buffer (with 1× TAE and 0.1% SDS) for 50 min at a voltage of 100 V and at 4°C. Finally, the proteins were transferred onto an immunoblot membrane for subsequent immunoblotting (50).

### Confocal imaging

HeLa or PK-15 cells were seeded into an eight‐well chamber slide (ibidi; 80826) and fixed with 4% paraformaldehyde at room temperature for 20 min. After washing with PBS, cells were permeabilized with 100% methanol at −20°C for 20 min, then blocked with 2% BSA in PBS for 1 h at room temperature, followed by incubation with relevant primary antibodies at 4°C overnight. The following day, cells were washed with PBST three times and incubated with an appropriate secondary antibody. Then, cells were washed with PBST three times and stained with DAPI (Invitrogen) at room temperature for 10 min. Images were acquired under Nikon laser scanning confocal microscope (C2plus) and analyzed using NIS‐Elements software.

### Luciferase assays

HEK293T cells cultured in 12-well tissue culture plates were transfected with 400 ng of IFN-β driving luciferase (firefly) plasmid, the internal control TK‐*Renilla* luciferase (*Renilla*) reporter plasmid, and relevant individual plasmids using PEI. At 24 h post-transfection, the cell layers were washed with PBS and lysed with 1× Passive Lysis buffer (Promega; E194A) for 15 min. Finally, the luciferase activity was estimated using the Dual-Luciferase Reporter Assay System (Promega; E1980) according to the manufacturer’s protocol. The values indicate the firefly luciferase activity normalized to the *Renilla* luciferase activity. On the other hand, the 293-Dual hSTING-A162 cells and QUANTI-Luci were used to estimate the 3× Flag cGAS or cGAMP-induced luciferase activity on hSTING with or without B175L-Flag.

### Enzyme-linked immunosorbent assay (ELISA)

ELISA was performed to detect the secreted pro-inflammatory cytokines in culture supernatants. Commercial kits for porcine IFN-β (CUSABIO, CSB-E09890p) and IL-6 (CUSABIO, CSB-E06786p) were used for the analysis according to the manufacturer’s instructions.

### *In vitro* 2′3′-cGAMP binding assay

The protein/2′3′-cGAMP-Biotin conjugate or protein/2′3′-cGAMP-Cy5 conjugate reactions were set up in 1.5-mL microcentrifuge tubes. Per 50-µL reaction, we added 2 µL of 2′3′-cGAMP-Biotin or 2′3′-cGAMP-Cy5 (1 mM), 5 µL of 10× reaction buffer (100 mM Tris, 500 mM KCl, and 10 mM DTT), and 0.5 µL of 200 mM EDTA (pH = 8), increasing concentrations of glycine-purified proteins, and 10 µg of STING-Strep and 1–10 μg of B175L-Flag, and UltraPure DNase/RNase-Free distilled water (Invitrogen) as required. Microcentrifuge tubes were then incubated for 2 h at 37°C on a rotter. Thereupon, the samples’ volume was increased from 50 μL to 800 µL by adding RIPA (50 mM Tris–HCl, 150 mM NaCl, 0.5% sodium deoxycholate, 1% IGEPAL). To pull-down protein/2′3′-cGAMP-Biotin or protein/2′3′-cGAMP-Cy5 conjugate reactions, 100 µL of Dynabeads M-280 Streptavidin (Invitrogen; 11205D) was added to relevant microcentrifuge tubes with biotinylated 2′3′-cGAMP and 2 µL of anti-Strep antibody for protein/2′3′-cGAMP-Cy5 conjugate. After overnight incubation at 4°C on a rotter, Dynabeads were separated with 4,000 rpm centrifugation for 5 min and washed with TBST (gentle vortex for 1 min, three cycles). On the other hand, samples containing protein-2′3′-cGAMP-Cy5 interactions were isolated from protein A/G PLUS‐Agarose. Finally, all bound proteins were purified by performing protein elution using a 100 mM glycine buffer solution at pH 2 to 2.5 (Santa Cruz Biotechnology). The resulting eluted fraction was subsequently neutralized using a 500 mM NH_4_HCO_3_ solution, and the eluted fractions were then subjected to SDS-PAGE.

### Mass spectrometry

The sample preparation was done as previously optimized (51). Briefly, HEK293T cells transfected with B175L-Strep or control were harvested at 36 h post-transfection. Cell lysates were pulled down with Strep-Tactin Sepharose resin overnight at 4°C. After washing the resin with PBS and lysis buffer, the resin-bound proteins were separated with elution buffer (100 mM Tris–HCl, 150 mM NaCl, 1 mM EDTA, and 2.5 mM desthiobiotin); the eluted proteins were further concentrated using an Amicon Ultra-0.5 (10K cutoff) centrifugal filter (Merck Millipore; UFC501096). Four to fifteen percent of NuPAGE gels (Invitrogen; NP0323PK2) were used for broad molecular weight protein separation, followed by silver staining (52). Next, the protein bands present in the gel were subjected to mass spectrometry analysis. Finally, the B175L-unique mass spectrometry results were filtered against the proteins of the control and checked for specific bound proteins whose importance is associated with IFN-I pathway. Among thousands of proteins, the STING (UniProt: Q86WV6) was selected for further studies, and that selection was dependent upon the biological relevance in the context of immunology, STING-related previous findings, and experimental feasibility.

### Statistical analysis

All graphs and statistical analyses were carried out with the GraphPad Prism software, version 6, for Windows. The data are presented as means and standard deviations (SD) for at least three independent experiments. At each time point, an unpaired *t*-test was used to compare the control and treatment groups. *P* values of <0.05, <0.01, <0.001, or <0.0001 were regarded as significant.

## SUPPLEMENTAL MATERIAL

Supplemental material jvi.00795-23-s0001.docx Fig. S1 and S2; Tables S1 and S2.

ASM does not own the copyrights to Supplemental Material that may be linked to, or accessed through, an article. The authors have granted ASM a non-exclusive, world-wide license to publish the Supplemental Material files. Please contact the corresponding author directly for reuse.
