# Supplementary material for: African swine fever virus B175L inhibits the type I interferon pathway by targeting STING and 2′3′-cGAMP
Source: J Virol. 2023 Oct 30;97(11):e00795-23. doi: 10.1128/jvi.00795-23 (PMC10688321; doi:10.1128/jvi.00795-23)
Supplement: Supplemental material — Fig. S1 and S2; Tables S1 and S2. [file jvi.00795-23-s0001.docx]

**Supplementary Figures**

**
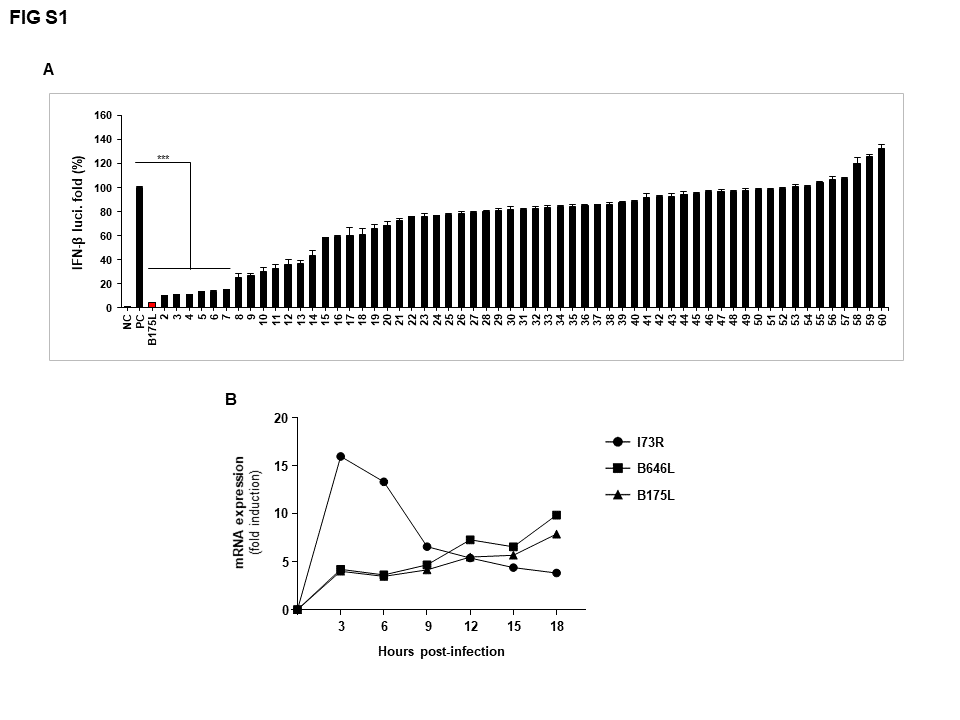
**

**FIG S1 The screening result of the ASFV gene library based on IFN-β luciferase activity**

(A) STING-Flag, TK Renilla luciferase, and IFN-β firefly luciferase promoter plasmids were transfected into HEK293T cells with or without ASFV genes (pIRES-Flag cloned sixty ASFV genes: GenBank: FR682468.1). After 24 h post-transfection, the IFN-β promoter activity was assessed using the dual-luciferase reporter assay system. (B) Primary PAM cells were infected with 1.0 MOI of ASFV (Korea/wildboar/Hwacheon/2020-2287). The cell pellets were harvested at 0, 3, 6, 9, 12, 15, and 18 hours post-infection (hpi). Subsequently, the extracted RNA was subjected to qRT-PCR analysis. The data represent at least three independent experiments with similar results, and the values are expressed as means and SD for three biological replicates. Student's t-test: *, P < 0.05; **, P < 0.01; ***, P < 0.001; ****, P < 0.0001; ns, not significant.

**
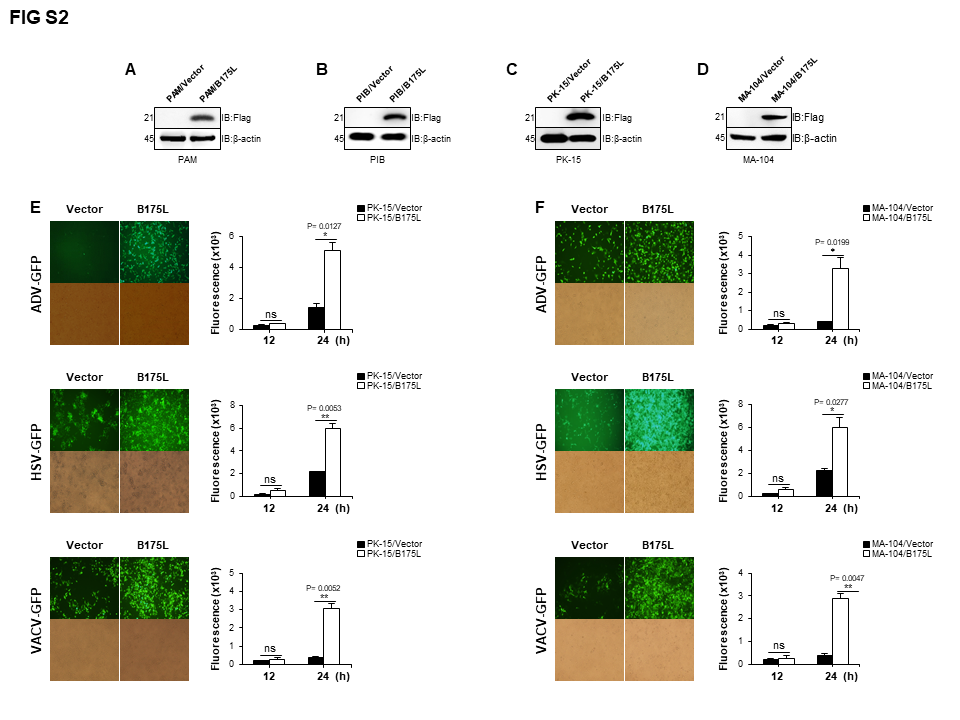
**

**FIG S2 Protein expressions in cell lines and GFP expressions after virus infection**

(A-D) Expression of B175L-Flag in stable PAM cells, stable PIB cells, PK-15 cells, and stable MA-104 cells. (E & F) PK-15 cells transiently expressing B175L-Flag or stable MA-104 cells infected with ADV-GFP, HSV-GFP, or VACV-GFP (MOI = 1.0). The GFP images were captured at 24 hpi using fluorescence microscopy and quantified at 12 and 24 hpi using the fluorescence modulator. Protein sizes are expressed in kilodaltons (kDa). All the immunoblot data are representative of at least two independent experiments, each with similar results. The values provided represent the means and standard deviations (SD) of three biological replicates, and these results are consistent across at least two independent experiments. Student's t-test: *, P < 0.05; **, P < 0.01; ***, P < 0.001; ****, P < 0.0001; ns, not significant.

**TABLE S1: List of primers used to generate STING mutations**

| **Mutation** | **Forward primer** | **Reverse primer** |
| --- | --- | --- |
| STING  R238A | GCATCAAGGATGCGGTTTACAGCAACAGCATC | GATGCTGTTGCTGTAAACCGCATCCTTGATGC |
| STING  Y240A | GCATCAAGGATCGGGTTGCCAGCAACAGCATC | GATGCTGTTGCTGGCAACCCGATCCTTGATGC |
| STING  N242A | GATCGGGTTTACAGCGAAAGCATCTATGAGCTTC | GAAGCTCATAGATGCTGGCGCTGTAAACCCGATC |
| STING  E260A | CGGGCACCTGTGTCCTGGCGGCGTACGCCACCCCCT | AGGGGGTGGCGTACGCCAGGACACAGGTGCCCG |
| STING R238A+Y240A | GCATCAAGGATGCGGTTGCCAGCAACAGCA | TGCTGTTGCTGGCAACCGCATCCTTGATGC |

**TABLE S2: List of primers used for real-time PCR**

| **Target gene** | **Forward primer** | **Reverse primer** |
| --- | --- | --- |
| B175L | ATGGAAACTAATTGTCCTAATATTT | TTAAATAAAAATGTGTTTGGAAGAA |
| pIFN-β | AAATCGCTCTCTGATGTGT | TGCTCCTTTGTTGGTATCG |
| pIFN-γ | CCATTCAAAGGAGCATGGAT | ATCCATGCTCCTTTGAATGG |
| pIL-6 | CACCGGTCTTGTGGAGTTTC | GTGGTGGCTTTGTCTGGATT |
| pIL-1β | GGGACTTGAAGAGAGAAGTGG | CTTTCCCTTGATCCCTAAGGT |
| pTNFα | TCACAGGGCAATGATCCC | GGGATCATTGCCCTGTGA |
| pMCP-1 | CAGAAGAGTCACCAGCAGCA | TCCAGGTGGCTTATGGAGTC |
| pMX-1 | TAGGCAATCAGCCATACG | GTTGATGGTCTCCTGCTTAC |
| pISG15 | AAATCGCTCTCCTGATGTGT | TGCTCCTTTGTTGGTATCG |
